# Saving fall-injured older adults from depressive symptoms: The mediating role of social participation

**DOI:** 10.1093/geroni/igab046.3142

**Published:** 2021-12-17

**Authors:** Yalu Zhang, Lei Zhang, Jiling Sun, Xinhui Zhang, Jingjing Sun, Xinming Song, Gong Chen

**Affiliations:** Peking University, Beijing, Beijing, China (People's Republic)

## Abstract

Falls are the second primary cause of unintentional injury deaths globally. Prior studies found that fall incidences are associated with depressive symptoms among older adults, which could reversely lead to repeated fall incidences. However, few have investigated the role of social interventions in saving fall-injured older adults from experiencing depressive symptoms among older adults. Using the Chinese Health and Retirement Longitudinal Study (CHARLS) 2011-2018 data and multiple levels of fixed-effect analysis, this study examined the potential mediating role of social participation in alternating the effect of fall injuries on depressive symptoms. For the first time, this study specified the fall-injured older adults among those who had fall incidences. It also implemented the current literature by removing the bias caused by unobservable confounding variables at provincial and city levels. The descriptive results show that 22.2% and 20.6% of rural (n=4,972) and urban (n=3,258) older adults (65+), respectively, experienced fall incidences, among whom 45.1% needed one or more times of medical treatment. The fixed-effect results show that for urban older adults, social participation accounted for partial effects (17.2%) of fall injuries on their depressive symptoms. For rural older adults, fall injuries are significantly associated with more depressive symptoms, but social participation no longer functions as the mediator. Findings from this study emphasize the necessity of collecting efforts from multiple levels to improve the social engagement of urban older adults who had fall injuries. Future studies could further specify what types of social participation would be more helpful in buffering the intervention effects.

